# A silent outbreak due to *Klebsiella pneumoniae* that co-produced NDM-1 and OXA-48 carbapenemases, and infection control measures

**DOI:** 10.22038/IJBMS.2019.35269.8400

**Published:** 2020-01

**Authors:** Yucel Duman, Yasemin Ersoy, Nafia Canan Gursoy, Sibel Altunisik Toplu, Baris Otlu

**Affiliations:** 1 Medical Microbiology Department, Inonu University Medical Faculty, Malatya, Turkey; 2 Infections Disease Medical Microbiology Department, Inonu University Medical Faculty, Malatya, Turkey

**Keywords:** Carbapenemase, Klebsiella pneumoniae, NDM, OXA-48, Outbreak

## Abstract

**Objective(s):**

Infections due to carbapenemase-producing *Klebsiella pneumoniae* are associated with high morbidity and mortality. In this study, we report a hospital outbreak due to co-producing OXA-48 and NDM-1 *K. pneumoniae* clone. The aim of the study is to investigate the clonal relationship of strains, risk factors of outbreak and infection control measures.

**Materials and Methods:**

Once an outbreak was suspected at the end of December 2017 in our intensive care unit (ICU), carbapenem resistance *K. pneumoniae* identified in patients’ specimens. An outbreak analysis was begun to determine the risk factors and dissemination of the cases. A case-control study was conducted to determine the risk factors. To control the outbreak; tight contact prevention, good clean-up the medical devices and hospital environment, were done. Staff training programs such as hand hygiene, disinfection, wearing aprons, good cleaning were created. Carbapenem resistance genes determined by PCR. Clonal relationships of strains investigated by PFGE.

**Results:**

We investigate 21 carbapenem-resistant *K. pneumonia* strains. Nine of them were found co-produced NDM-1 and OXA-48, 11 strains produced OXA-48, and one strain produced NDM-1. Seven strains of co-producing NDM-1 and OXA-48 were found clonally related with PFGE. We could not determine any risk factor except rectal colonization in the case-control study.

**Conclusion:**

The interventions that successfully controlled this outbreak were hand hygiene, tight contact prevention, good clean-up of the hospital environment and medical devices. As a result, we believe that it would be beneficial to take infection control measures to prevent the spread of these strains to the community and hospital settings.

## Introduction

*Klebsiella pneumoniae* is a significant cause of nosocomial infections (1). It is reported that *K. pneumoniae* is responsible for the 18% to 31% of all nosocomial infections, and 3% to 17% of community-acquired infections. In various studies, the mortality due to *K. pneumonia* is reported between 30% to 54% in intensive care units (2-4).

Carbapenems are the last group of antimicrobials active against multidrug-resistant (MDR) Gram-negative organisms. They are particularly indispensable in various life-threatening infections caused by extended-spectrum (ESBL) and AmpC ß-lactamase-producing strains. Furthermore, carbapenems have excellent tissue distribution and lower side effects than polymxyins and tigecycline which can be used against *K. pneumoniae*. Also, carbapenems may be used more safely in children’ and adults’ treatments (1, 5). Until a decade ago, carbapenem resistance was rarely reported in *K. pneumonia* strains. However, carbapenemase-producing *K. pneumoniae *(CPKP) isolates emerged and disseminated worldwide, principally producing the oxacillinase-48 (OXA-48) like, New-Delhi metallo-ß-lactamase (NDM)-type and *K. pneumoniae *carbapenemases (KPC)-type enzymes (6). Infections due to CPKP strains are reported very frequently and represent a significant clinical concern. CPKP-related infections are associated with high morbidity and mortality rates due to limited treatment options. The clinically most important carbapenemase genes in the CPKP are Ambler class A (KPC type), B class (NDM, VIM and IMP types) and D class (OXA-48-like). OXA-48 is often in Turkey and North Africa, KPC is in United States, Greece and Italy, NDM is often detected in India (1, 6).

NDM producing strains can spread from water sources, from a contaminated environment, from animal to humans, and also human to human. NDM-producing strains also carry resistance genes against virtually all beta-lactamases, aminoglycosides, quinolones, nitrofurantoin and sulphonamides. Furthermore, due to NDM-encoding multiple chromosomal and plasmid-derived resistance genes in NDM-producing strains, this resistance can be easily spread (1, 7). 

The first* K. pneumoniae* infection due to co-producing OXA-48 and NDM-1 is reported in 2013 in Morocco (8). Since then, a few studies have been reported. However, there are not sufficient epidemiological, clinical and infection control measures report on co-producing OXA-48 and NDM-1 *K. pneumoniae *infections.

The study was performed due to the increase of the carbapenem resistant *K. pneumoniae* (CRKP) strains in the 3-month period from October to December 2017. We aimed to investigate the clonal relationship between the co-producing OXA-48 and NDM-1 *K. pneumoniae* strains, risk factors of outbreak and infection control measures. 

## Materials and Methods

***Setting and definition***

The outbreak occurred in a 1350-bed capacity university hospital in Eastern Turkey. There are 16 intensive care units’ (ICU) and 253 ICU beds in the hospital. We choose all patients who were infected with CRKP strains. Once an outbreak was suspected at the end of December 2017, an outbreak analysis was begun to determine the risk factors and dissemination of the cases. Retrospective and prospective analysis were undertaken. A case-control study was conducted to determine the risk factors. Data on patients, including age, gender, underlying diseases, invasive procedures and other risk factors were collected from electronic records, patient files and from the medical personnel. Also, infection control measures were applied immediately. Some samples were collected from suspected areas to identify the source of the outbreak and rectal carriage was investigated in the related ICUs.

***Identification and susceptibility***

*K. pneumoniae* strains were identified by conventional methods and Maldi-tof MS (BioMérieux, France) from the clinical specimens. *In-vitro* antimicrobial susceptibility tests of these strains were determined on the basis of criteria of Clinical and Laboratory Standards Institute (CLSI) by Kirby-Bauer disk diffusion method and automated system (Microscan, Beckman, USA) (9). *Escherichia coli* ATCC 25922 strain was used as a standard.

***Detection of carbapenemase genes***

The presence of carbapenemase genes were screened by in-house PCR using the primer sets as follows; OXA-48-F-TGTTTTTGGTGGCATCGAT and OXA-48-R-GTAAMRATGCTTGGTTCGC for *bla*_OXA-48_, NDM-1-F-TTGGCCTTGCTGTCCTTG and NDM-1-R-ACACCAGTGACAATATCACCG) for *bla*_NDM-1_, KPC-F-TCGCTAAACTCGAACAGG and KPC-R- TTACTGCCCGTTGACGCCCAATCC) for *bla*_KPC_*,* VIM-F-GATGGTGTTTGGTCGCATA and VIM-R- CGAATGCGCAGCACCAG) for *bla*_VIM_ and IMP-F-GGAATAGAGTGGCTTAAYTCTC and IMP-R- CCAAACYACTASGTTATCT) for *bla*_IMP _(10, 11). For this purpose, firstly DNA extraction of strains was performed with a column-based DNA isolation kit (DNA mini kit, Qiagen, Germany). The PCR run was performed using the GeneAmp PCR System 9700 device (Applied Biosystems) and amplicons were photographed with UV illumination after electrophoresis. Amplification conditions; Following initial denaturation step for 3 min at 94 ^°^C, 35 cycles of denaturation at 94 ^°^C for 30 sec, annealing at 60 ^°^C for 30 sec, and extension at 72 ^°^C for 1 min, and a final elongation step at 72 ^°^C for 10 min. 

***Molecular typing***

The clonal relationships of these strains were investigated by pulse-field gel electrophoresis (PFGE) according to a previously described protocol (12). PFGE restriction pattern was obtained on a CHEF DR II-apparatus (Bio-Rad Labs, Belgium) using XbaI (Promega Co.) as a restriction enzyme. The electrophoresis conditions were 14 ^°^C at 120 V for 20 hr, with pulse time ranging from 5 to 30 sec. Dendrogram construction was performed using the Dice coefficient and unweighted-pair group method using average linkages (UPGMA) clustering. Isolates were accepted as being the same pulse type if the value of the Dice coefficient of similarity was >95%. Gels were stained with ethidium bromide and photographed under UV light transilluminator (Gel Logic 2200 Imaging System, Kodak, NY) and analyzed with GelCompar V.6.6 software (Applied Maths, Belgium).

## Results

**Figure 1:**
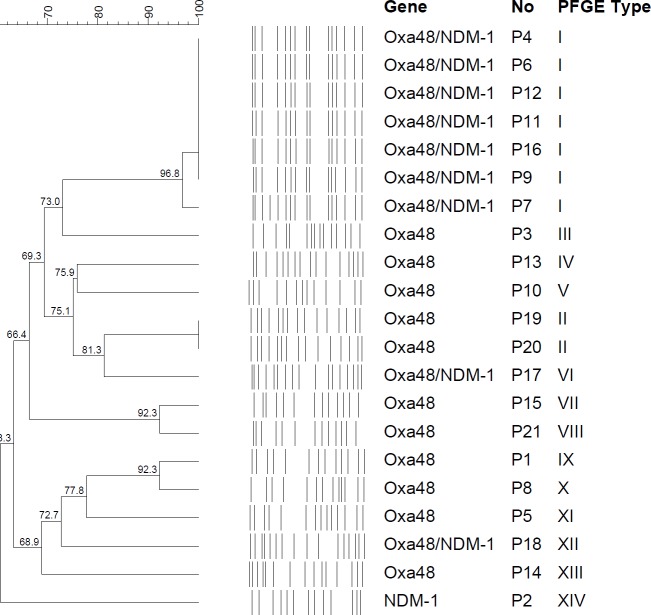
Pulse-field gel electrophoresis (PFGE) of the strains

In this study, we investigate 21 CRKP strains which were isolated in our hospital ICU during the 3 months period from October to December 2017. All strains were resistant to carbapenems (imipenem, meropenem and ertapenem), fluoroquinolones, trimethoprim-sulfamethoxazole and ß-lactam antibiotics. Only 3 strains susceptible to aminoglycosides and all strains were susceptible to colistin and tigecycline. Nine of 21 strains were found to co-produce NDM-1 and OXA-48, 11 strains produced OXA-48, and one strain produced NDM-1. Seven of nine co-producing NDM-1 and OXA-48 strains were found clonally related with PFGE (Figure 1). Additionally, 2 of 12 OXA-48 producing strains we found clonally related.

We recognized that the first patient was admitted to our hospital on October 10, 2017. The NDM-1 positive *K. pneumonia* strain was isolated from this immigrant child within two days after hospitalization. The patient was determined as an index case. Before the index case, we determined only OXA-48 producing *K. pneumonia* strains in our hospital. After the hospitalization of the index case, we isolated NDM-1 and OXA-48 co-producing *K. pneumonia* strain from the patients. The patients’ hospitalizations and *K. pneumonia* isolation date, specimen, treatment, and outcome are shown in Table 1. The risk factors such as radiologic invasive procedure, orthopedic surgical procedure, intra-abdominal surgical procedure, carbapenem use, invasive catheterization, mechanic ventilation, underlying diseases for this co-producing NDM-1 and OXA-48 positive *K. pneumoniae* outbreak was investigated, yet we could not determine any risk factor except rectal colonization in the case-control study (Table 2). Rectal colonization was found in five patients.

***Infection control interventions***

Infection control measures such as staff training, tight contact prevention, good clean-up of the hospital environment and medical devices (aspirator heads, ventilator surfaces, *etc*.) in ICUs were taken quickly. Also hand hygiene, cleaning, and disinfection training programs (such as good cleaning of the patient’s surrounding, wearing clean aprons during the dispensing of clean materials, wearing aprons and preparing clean materials for the preparation of new patient beds) were created to educate staff. During the staff work, observation and feedback were taken by the committee nurse. Further, awareness created in staff by training about the importance of CRKP and CPKP infections. After infection control measures CRKP strains were not observed in the ICU since January to May 2018.

## Discussion

*K. pneumoniae* is an important nosocomial pathogen that can cause infections resulting in severe morbidity and mortality. In hospital settings, infections due to *K. pneumoniae* usually occurs with multidrug resistance (MDR) strains. Therefore, it causes, treatment difficulties and high mortality rates. While the treatment of nosocomial *K. pneumoniae* infections is often performed with carbapenem group antibiotics. Thus the resistance rates of carbapenems have been started to increase rapidly in parallel with the use of carbapenem. In fact, this is directly related to the selection of carbapenem resistant strains during treatment (13, 14).

Over the years, more than 350 beta-lactamases and more than 26 carbapenemase enzymes have been identified in Gram-negative bacteria (1, 15). Carbapenemase-producing *Enterobacteriaceae* was first reported in 1993. The mechanism of resistance to carbapenems is mostly associated with beta-lactamases enzymes transmitted through chromosomes or plasmids; Verona integron-encoded metallo-beta-lactamase (VIM), KPC, NDM-1, OXA-48 and the imipenem-hydrolyzing beta-lactamase (IMI/IMP). Carbapenemases have become a major problem worldwide now. These carbapenemases are a growing concern for global health because they are resistant to β-lactam antibiotics and other classes of antibiotics such as aminoglycosides, fluoroquinolones, and co-trimoxazole. For this reason, the possibility of treating infections due to MDR strains is diminishing (1, 6, 15).

Turkey is in an endemic situation for OXA-48 producing *K. pneumonia* and it is reported frequently. However, NDM-1 producing *K. pneumonia* strains have recently been reported. In Turkey, OX-48 was first described in 2003 in *K. pneumoniae* strains (16). Turkey may be one of the main reservoirs of OXA-48-producing *K. pneumoniae* strains. Since 2003, the endemic spread of these bacteria has been reported in countries such as Turkey, Morocco, Libya, Egypt, Tunisia, and India (6, 16). Between 2004 and 2005 Aktas *et al.* (17) found OXA-48 producing *K. pneumoniae* strains from 2 patients who had a long hospital stay and meropenem use. In 2008 Carrer *et al. *(18) reported an OXA-48 producing *K. pneumoniae* outbreak in a hospital in Istanbul. Us *et al. *(19) reported that OXA-48 positivity of *K. pneumoniae* strains is 26.9% in Turkey. 

Since 2008, NDM producing *K. pneumoniae* strains have spread rapidly in many countries. NDM producing *K. pneumoniae* strains are accepted endemic in countries including India, Pakistan and Bangladesh (20). In Turkey first spread had been reported sporadically in 2015 (21). Center for Disease Control and Prevention; the spread of *Enterobacteriaceae* strains producing carbapenemase all over the world is described as an immediate threat and the World Health Organization reports the development of new antimicrobials as a critical need (22, 23). 

In the report of European survey of carbapenemase-producing *Enterobacteriaceae* (EuSCAPE) working group which was formed in 2012 by European Centre for Disease Prevention and Control (ECDC); Turkey is classified in phase 5 country groups in terms of the OX-48 producing *K. pneumoniae* (0: cases have been reported; 1: Sporadic 2: One hospital outbreaks 3: Regional expansion 4: Interregional extension 5: endemic). However, for NDM in 2013 Turkey is classified in phase 1 country groups, but in 2015 taken in phase 3. In addition, the report emphasizes that NDM-1 producing *Enterobacteriaceae* strains have been increasing from 2013, particularly in health facilities near the Syrian border (7).

The first co-producing OXA-48 and NDM-1 *K. pneumoniae* strain was identified in 2015 in Turkey (24). In 2017, the first NDM-1 producing *K. pneumoniae* outbreak has been reported (25). According to our information, we will be the second study to report an outbreak of co-producing OXA-48 and NDM-1 *K. pneumoniae*. We identified co-producing OXA-48 and NDM-1 in nine *K. pneumoniae* strains that caused the outbreak in our study. In addition, there is no other publication that shows the origin of NDM-1 producing *K. pneumoniae* strain, which causes an outbreak, according to our knowledge. In our study, there is strong evidence that the NDM-1 gene region is transmitted from a migrant patient to OXA-48 positive *K. pneumoniae* strains in our hospital. In studies, it is reported that inter-country travel of patients’ may be a trigger for the international spread of carbapenemase-producing *K. pneumoniae *(23).

Despite all the infection control measures taken to prevent infections caused by MDR microorganisms, carbapenem resistant *K. pneumoniae* infections are reported increasingly worldwide. As other hospitals in our country, carbapenem resistance is found with OXA-48 type beta-lactamases and in our hospital. In our study, we observed that carbapenem resistant *K. pneumoniae* strains increased in our hospital during the three month period from October to December 2017. We identified nine co-producing OXA-48 and NDM-1 *K. pneumoniae *strains, and seven of them are the same clone. When we examine the last ten year period in our hospital; we determine OXA-48 producing *K. pneumoniae* strain since last 3 years, but we did not identify NDM-1 producing strain. In the outbreak analysis, we investigated risk factors for co-producing NDM-1 and OXA-48 positive *K. pneumoniae*, but we did not determine any risk factor in the case-control study, however, rectal colonization was found in five patients. Due to the widespread presence of OXA-48 producing strains in our hospital, we believe that a strain that also acquired the NDM-1 gene locus causes an outbreak in the ICU. 

As of December 2018, infection control measures had been increased in our hospital, considering that it might be an outbreak. Personnel training programs for hand hygiene and environmental and equipment cleaning were organized and new procedures for equipment and environmental clean-up prepared. The isolation of these patients is provided. As the January to May no new carbapenem resistant *K. pneumoniae* infections had been observed. This shows us that applying the hand hygiene and equipment cleanliness rules, creating awareness in staff is very important to prevent the spread of infection.

These results show that practice guidelines are needed to prevent hospitals from facing uncontrolled outbreaks, and that staff training and awareness needs to be done on a regular basis. In a recent report, an OXA-48 producing *K. pneumoniae* outbreak was reported at a German University Hospital. Hygiene regulations necessity is remarked for various nosocomial environments, including endoscopic instruments, aprons, and gloves (26). Examination of the epidemiological characteristics of OXA-48 producing *K. pneumoniae* outbreak in an ICU in France revealed that the outbreak was due to the environmental continuity of OXA-48 producing *K. pneumoniae* for 20 months. This report emphasizes the importance of early environmental screening to eradicate the transmission of CPKP (27).

**Table 1:** Patients’ hospitalizations date, *Klebsiella pneumonia* isolation date, specimen, treatment and outcome

**Patients**
	**Age**	**Sex**	**PFGE type**	**OXA48**	**NDM-1**	**Isolation date of ** ***K. pneumoniae***	**Hospitalization date of patients**	**Hospitalization unit**	**Specimen**	**Treatment**	**Outcome**
P1	19	M	IX	P	N	10/11/2017	09/13/2017	ICU	Blood	Mem+Ak	Dead
P2	1	M	XIV	N	P	10/12/2017	10/10/2017	ICU	Urine	Mem+ Tig	Referred
P3	36	F	III	P	N	10/23/2017	10/04/2017	ICU	Wound	Tig+Tzp	Cured
P4*	36	M	I	P	P	10.25/2017	10/09/2017	ICU	Wound	Col+Mem+ß lactam	Cured
P5	35	F	XI	P	N	11/03/2017	10/16/2017	ICU	Blood	Imp+Ak+ Tig	Dead
P6*	31	M	I	P	P	11/04/2017	10/08/2017	ICU	Wound	Col+ Tig + Imp	Dead
P7*	72	F	I	P	P	11/06/2017	10/18/2017	ICU	Endotracheal	Mem+ Tig +Ert	Dead
P8	70	F	X	P	N	11/07/2017	10/18/2017	ICU	Endotracheal	Mem+Ert	Dead
P9*	39	F	I	P	P	11/09/2017	10/31/2017	ICU	Blood	Mem+Ak+Col	Dead
P10	73	M	V	P	N	11/10/2017	10/02/2017	ICU	Urine	Tzp+Cip	Cured
P11*	71	M	I	P	P	11/15/2017	10/19/2017	ICU	Endotracheal	Mem+ Tig	Dead
P12*	53	M	I	P	P	11/16/2017	11/10/2017	ICU	Blood	Tig+Mem+Ert	Dead
P13	61	F	IV	P	N	11/28/2017	11/10/2017	ICU	Blood	Col+ Tig +Imp	Dead
P14	20	M	XIII	P	N	11/29/2017	11/09/2017	ICU	Endotracheal	Col+Mem+ Tig	Cured
P15	39	F	VII	P	N	12/01/2017	10/19/2017	ICU	Endotracheal	Tig+ Imp	Cured
P16*	74	M	I	P	P	12/15/2017	11/25/2017	ICU	Endotracheal	Col+Mem+ Tig	Dead
P17*	24	F	VI	P	P	12/18/2017	12/11/2017	ICU	Endotracheal	Mem+Ert	Dead
P18*	76	F	XII	P	P	12/19/2017	12/02/2017	ICU	Endotracheal	Mem+ Tig	Dead
P19	87	M	II	P	N	12/20/2017	08/16/2017	ICU	Blood	Mem+ Tig	Dead
P20	61	M	II	P	N	23/12/2017	09/13/2017	ICU	Endotracheal	Mem+Col	Dead
P21	64	F	VIII	P	N	26/12/2017	12/21/2017	ICU	Wound (OM)	Imp+Tig	Dead

*:co-producing OXA-48 and NDM-1 *K. pneumoniae*, N: negative, P: positive, Mem: Meropenem, Imp: Imipenem, Tig: Tigecycline, Ert: Ertapenem, Col: Colistin, Ak: Amikacin, Tzp: Piperacillin-Tazobactam, Cip: Ciprofloxacin, OM: Osteomyelitis

**Table2:** Analized of risk factors for co-producing NDM-1 and OXA-48 positive *K. pneumoniae*

**Parameters**	**Cases** **n: 8**	**Controls** **n: 16**	
Underlying diseases	5	11	*P*-value >0.5
Radiologic invasive procedure	4	9	*P*-value >0.5
Orthopedic surgical procedure	3	1	*P*-value >0.5
Intra-abdominal surgical procedure	2	7	*P*-value >0.5
Carbapenem use	4	7	*P*-value >0.5
Invasive catheterization	5	14	*P*-value >0.5
Mechanic ventilation	6	13	*P*-value >0.5
Rectal colonization	5	0	*P*-value<0.001

## Conclusion

We determined that; co-producing OXA-48 and NDM-1 *Klebsiella pneumoniae* led to an outbreak in ICU in our hospital. The interventions that successfully controlled this outbreak were hand hygiene, prevention of patient transfer to other units, preparing new procedures for equipment and environmental clean-up and creating awareness in staff. In this context, it is necessary to be more careful in order to prevent the spread and selection of CPKP strains in hospitals. It is possible that CPKP strains may be the spread to the community from hospital settings. For this reason, the fact that such bacteria can be spread during the transfer of patients to different centers of health must be known. As a result, we believe that it would be beneficial to take infection control measures to prevent the spread of these strains to the community and hospital settings.
